# Saccadic eye movements differentiate functional cognitive disorder from mild cognitive impairment

**DOI:** 10.1177/03010066251359215

**Published:** 2025-07-29

**Authors:** Thomas D. W. Wilcockson, Sankanika Roy, Trevor J. Crawford

**Affiliations:** 5156 Loughborough University, UK; 156756 Leicester Royal Infirmary, UK; 4396 Lancaster University, UK

**Keywords:** functional cognitive disorder, functional neurological disorder, antisaccade task, prosaccade task, mild cognitive impairment

## Abstract

Functional Cognitive Disorder (“FCD”) is a type of Functional Neurological Disorder characterised by subjective cognitive complaints not fully attributable to brain injury, disease, or other neuropathological or psychiatric conditions. FCD is a cognitive impairment but does not necessarily “convert” to cognitive decline. However, FCD is common in Memory Clinics worldwide, and currently there is a lack of tests to objectively assess FCD. Establishing whether memory complaints are functional or not is vital for clinicians and objective tests are required. Previous research indicates that early-stage Alzheimer's disease can be differentiated from healthy individuals by antisaccade eye-movement. Therefore, eye movements may be able to objectively ascertain whether self-reported memory complaints are functional in nature. In this study, FCD participants were Memory Clinic patients who self-reported memory complaints but showed internal inconsistency regarding memory issues on memory tests. Participants with FCD were compared to Mild Cognitive Impairment (MCI) patients and healthy controls (HC) on antisaccadic and prosaccade eye movement tasks. The parameters obtained were reaction-time (RT) mean and SD and antisaccade error rate. MCI differed significantly from HC in antisaccade RT-mean, RT-SD, error-rate, and from FCD antisaccade RT-mean, RT-SD, and error-rate. FCD did not differ significantly from HC for antisaccade parameters. However, FCD differed significantly from HC for prosaccade RT-mean and RT-SD. MCI did not differ significantly from HC or FCD in prosaccade parameters. These results indicate that eye movement tasks could ultimately aid clinicians in the diagnosis of FCD. With additional research into sensitivity and specificity, eye movement tasks could become an important feature of memory clinics.

## Introduction

Functional Cognitive Disorder (FCD) is a type of Functional Neurological Disorder characterised by self-reported memory or cognition complaints not fully attributable to brain injury or disease (Silverberg & Rush, 2024; McWhirter et al., 2020). FCD can affect patients of all ages (Pennington et al., 2015), however, in older adults, it is sometimes confused with neurodegenerative diseases. Older adult patients may present to Memory Clinics because they are concerned about a perceived memory or cognitive deficits, which they believe may be related to cognitive decline or impending dementia. However, importantly, for patients with FCD these complaints may be internally inconsistent (Ball et al., 2020) and may not be supported by objective neuropsychological assessments, neuroimaging, or laboratory tests (Stone et al., 2015). FCD can resemble several cognitive disorders like dementia due to Alzheimer's or alcohol-related cognitive impairments. Diagnosis of FCD therefore requires careful consideration by healthcare experts who must differentiate it from other conditions with similar symptoms. Current diagnostic methods involve thorough interpretation of medical history, precise neuropsychological examinations, and sometimes diagnostic tests including CT, MRI, thus resulting in the utilisation of healthcare resources and a high direct and indirect cost (Stone et al., 2015). Often, clinicians consider FCD as being a “diagnosis of exclusion” or clinicians may confuse FCD with feigning or malingering (Van Patten & Bellone, 2023). Currently, the knowledge of specific diagnostic tools available to screen FCD is lacking (Cabreira et al., 2023). FCD cases constitute a significant portion of patients seen in Memory Clinics (Borelli et al., 2022). Clinicians working in these specialised settings are often faced with the daunting task of distinguishing between cognitive complaints that are functional and those that are indicative of an underlying neurodegenerative disease. The absence of clear diagnostic markers and the reliance on subjective reports make this a formidable challenge. To address this issue, there is an urgent demand for objective assessments that can assist in differentiating FCD from cognitive disorders attributable to brain injury or disease. By identifying the presence of FCD then patients could receive appropriate care.

Cognitive impairment, typically observed in neurodegenerative conditions such as Alzheimer's disease is associated with measurable deficits in cognitive domains characterised by impairments in memory, language, executive function, or visuospatial skills (Kirova et al., 2015). In contrast, individuals with FCD may experience subjective cognitive difficulties but may demonstrate internal inconsistency and there is a heterogeneity of presentation within FCD, which includes subjective cognitive complaint with objective impairment, subjective cognitive complaint with no objective impairment, and informant complaint of cognitive trouble with and without evidence of objective impairment. However, note that there is often a distinction between objective and subjective impairment (Pennington et al., 2015) and self-reported cognitive complaints in an individual who does not inherently signal the initiation of cognitive decline. Jessen et al. (2020) have reported that, intriguingly, only a minority of individuals with FCD progress to clinically confirmed dementia. Recent advances in neurocognitive research have opened up exciting possibilities for objective diagnostic tools in the assessment of cognitive complaints. One particularly promising avenue of investigation is the role of eye movements in distinguishing between functional and systemic cognitive complaints. The potential of eye movements as a diagnostic tool is supported by previous research, particularly in the context of Alzheimer's disease and Mild Cognitive Impairment (MCI). Wilcockson et al. (Wilcockson et al., 2019) reported that individuals with MCI or Alzheimer's disease could be differentiated from healthy controls based on antisaccade eye movement deficits. The results indicate that cognitive decline is associated with deficits in the inhibitory control of saccadic eye movements. This finding suggests that specific patterns of eye movements may serve as objective markers of cognitive impairment, and therefore, could potentially be applied to distinguish between functional and systemic cognitive complaints.

Antisaccade eye movements are voluntary eye movements that involve looking in the opposite direction of a suddenly presented distractor. The antisaccade task requires the individual to actively inhibit automatic responsive eye movements and instead look in the opposite direction. This task engages various cognitive processes, including inhibitory control, working memory, and top-down goal-directed behaviour (Munoz & Everling, 2004). The ability to perform antisaccades relies on intact executive functions, which encompass a range of higher-order cognitive processes involved in goal-setting, planning, and response inhibition. Deficits in executive functions are a common feature in neurodegenerative conditions such as Alzheimer's disease (Perry & Hodges, 1999). Therefore, antisaccade eye movements offer valuable insights into the integrity of cognitive processes and, by extension, the presence of functional or systemic cognitive issues.

FCD, like Functional Neurological Disorder (FND), is thought to have predisposing, precipitating, and perpetuating factors (Hallett et al., 2022). Individuals with FCD may have intact cognition and memory but may misattribute normal everyday memory lapses to neuropathology (Wakefield et al., 2018), especially in elderly people who may attribute normal ageing-related memory lapses to a fear of cognitive decline (Silverberg & Rush, 2024). Elderly people may ruminate and excessively focus on these perceived lapses in memory that could be driven by a heightened awareness of any cognitive difficulties. If this is the case, then conscious compensation of attentional resources may therefore occur as a coping mechanism to ensure that potential lapses are monitored and subsequently corrected. Although highly speculative, if individuals with FCD are acutely aware of their cognitive challenges, they might make conscious efforts to compensate for perceived lapses in attention. This compensation could involve strategies such as slowing down, focusing more deliberately on tasks, or allocating additional mental effort to maintain attention. Thus, conscious monitoring of attention may delay processing speed, but improve perceived accuracy. Therefore, in FCD, rumination can cause attentional lapses as more effort and conscious compensation is used to monitor own performance (Pennington et al., 2015; Stone et al., 2015). If this is the case, we would expect to find increased top-down control of attentional processes.

The antisaccade, is by nature, a top-down goal-directed saccadic task. The act of inhibiting a saccade is effortful and automatic processes cannot be relied upon. This type of saccade task is distinct from the prosaccade task, where instead participants are told to direct their eyes as quickly and as accurately toward a target, rather than away. Therefore, the prosaccade is a task that measures reflexive bottom-up movements of the eye. This distinction is important, as attentional processing speed has been hypothesised to be impaired in FCD (Teodoro et al., 2018). If FCD patients are consciously monitoring attention, then bottom-up reflexive eye movement may be impaired as a result of this. Therefore, it may be that FCD patients would demonstrate delayed prosaccade eye movements; however, as there are no issues attributable to brain injury or disease affecting eye movement inhibitory control, we would not expect a reflexive antisaccade deficit. Research regarding prosaccade performance in MCI groups is mixed (Opwonya et al., 2022), however, research typically indicates that MCI participants are not so readily impaired on this task due to its simplicity in directing attention. It seems that deficits on prosaccade tasks only exist when a participant hypothetically has slower processing speed as a result of increased control over attention (Teodoro et al., 2018). Therefore, if FCD patients are consciously monitoring attention, then bottom-up reflexive eye movement may be impaired. This would therefore be measured as a delayed prosaccadic eye movement. The antisaccade task itself requires cognitive control of eye movements (rather than reflexive) and would therefore not be negatively impaired by increased conscious monitoring of attention. If this is the case, then this distinct pattern of eye movements would indicate a specific eye movement profile for FCD and may aid clinicians in patient diagnosis.

The present study aims to investigate the utility of antisaccade and prosaccade eye movements in distinguishing individuals with FCD from those with MCI and healthy controls. The primary objective is to explore whether specific patterns of antisaccade and prosaccade eye movements can differentiate each participant group. Our first hypothesis posits that individuals with FCD, despite reporting memory complaints, will exhibit performance on antisaccade eye movement tasks comparable to healthy controls. This hypothesis is grounded in the assumption that, if the cognitive complaints in FCD are truly functional and not indicative of underlying neurodegenerative processes, then performance on tasks involving executive functions, such as the antisaccade task, should mirror that of individuals without cognitive complaints. Our second hypothesis predicts that individuals with FCD would demonstrate prosaccade deficits not present in either the MCI or healthy control groups as FCD has been previously observed to lead to more deliberate and controlled attention (Pennington et al., 2015; Stone et al., 2015). To test these hypotheses, a comprehensive assessment involving a sample of Memory Clinic patients with FCD, MCI, and a cohort of healthy controls was conducted. Participants underwent a battery of standardised neuropsychological tests to confirm their diagnostic classification and completed antisaccade and prosaccade eye movement tasks to evaluate their eye movement performance.

## Method

### Participants

Participants were men and women between the ages of 55 and 90, with at least 11 years of education and fluent English speakers. Of these, 22 were categorised as FCD, 31 had a diagnosis of MCI, and 72 were age-matched and education-matched cognitively healthy people to act as control participants (see Table 1). Control participants were recruited from the local community or were the spouse/partner of the FCD/MCI participants. All participants were white British or European. FCD and MCI participants were recruited through Memory Clinics in the National Health Service (NHS) where they had been referred following subjective memory complaints. The final sample size of 125 participants (FCD: 22, MCI: 31, Control: 72) was determined by a combination of factors. First, we aimed to recruit a sufficient number of participants within each group to ensure adequate statistical power for our analyses. Second, our sample size was constrained by the budgetary limitations of the grant, which influenced the recruitment and data collection efforts. Pre-screening of participants by a clinician ensured that all participants met the eligibility criteria of the study prior to our data collection. Note, there were no dropouts. However, not all participants were invited to participate in both antisaccade and prosaccade tasks due to time constraints. The number of each participants in each task can be found in Table 2.

**Table 1.** Descriptive statistics (SD) of participants including cognitive assessment (MoCA) scores for each group.

	Functional cognitive disorder	Mild cognitive impairment	Control participants	FCD – MCI	FCD – HC	MCI – HC
Age	68 (6.9)	71 (6.0)	69 (8.7)	.261	.809	.377
Sex (% male)	45.5%	35.5%	33.0%	.754	.999	.646
MoCA total score	27 (0.9)	21 (4.0)	27 (1.7)	<.001*	.802	<.001*
FCSRT – free recall	33.5 (4.9)	18.8 (7.3)	35.2 (6.1)	<.001*	.542	<.001*
FCSRT – total	47.2 (1.5)	44.8 (4.8)	47.6 (1.2)	.028*	.878	.001*
Education years	12.6 (4.2)	13.1 (4.0)	12.5 (2.7)	.821	.998	.715
Weekly alcohol units	3.4 (5.2)	4.8 (8.8)	7.3 (0.4)	.820	.166	.393

*Note*. Significant differences between groups are denoted by asterisk “*”. MoCA = Montreal cognitive assessment; FCSRT = Free cued selective reminding task, free recall, and total score.

**Table 2.** Means and standard deviation of pro and antisaccade performance in each group.

	FCD		MCI		HC		FCD – MCI	FCD – HC	MCI – HC
	*N*	*M*	*N*	*M*	*N*	*M*	*p*	*p*	*p*
Antisaccade RT mean (ms)	22	353 (55)	29	414 (81)	54	340 (85)	.004*	.494	<.001*
Antisaccade RT SD	22	86 (24)	29	105 (35)	54	79 (31)	.030*	.356	<.001*
Antisaccade error rate (%)	22	10 (11)	29	27 (30)	54	11 (12)	.022*	.666	.020*
Prosaccade RT mean (ms)	15	231 (50)	10	214 (23)	73	199 (42)	.257	.012*	.293
Prosaccade RT SD	15	57 (23)	10	54 (35)	73	40 (28)	.761	.022*	.142

*Note*. Significant differences between groups are denoted by asterisk “*”.

MCI participants received a clinical diagnosis following a full assessment with a dementia specialist. Those with a diagnosis of MCI met the following criteria: (1) subjective complaints of memory decline (reported by the person themselves or an informant); (2) objective memory or other cognitive impairment (considered when scores on standard cognitive tests were >1.5 SDs below age/education adjusted norms) with or without deficits in other cognitive domains; (3) intact daily-life activities (Petersen, 2004).

Those categorised as FCD met the Ball et al. (2020) criteria: (a) Symptoms of impaired cognitive function (subjective complaints of memory decline reported by the person themselves or an informant); (b) Clinical evidence of internal inconsistency (patient subjective cognitive decline was not supported by objective memory tests: ascertained by MoCA scores >26 and FCSRT free recall scores > 50% and total scores > 90%: see below); (c) Symptoms or deficit that are not better explained by another medical or psychiatric disorder (patients were screened for other medical or psychiatric disorders); and (d) Symptoms or deficit that cause clinically significant distress, or warrants medical evaluation (patients were seeking medical evaluation at a Memory Clinic); additionally, FCD patients had intact daily-life activities.

All participants were screened for visual acuity using the Snellen's chart and intact colour vision according to the Ishihara test (Ishihara, 1983). All potential participants were not eligible for the study if they had a previous history of head trauma, stroke, cardiovascular disease, active or past alcohol or substance misuse or dependence (participants reporting over 20 units of weekly alcohol consumption were also excluded), or any physical or mental health condition (including depression). Those with a global or specific learning disability were also not eligible to participate in the study. All participants had the capacity to consent to participation in the study and signed informed consent. Ethics’ Committee approval was granted by Lancaster University and NHS Health Research Authority (IRAS project ID: 81660).

### Stimuli and Tasks

Eye tracking assessment utilised the Antisaccade Task (AST) and Prosaccade Task (PST), while cognitive functions were evaluated using the Montreal Cognitive Assessment (MoCA) and the Free and Cued Selective Reminding Test with Immediate Recall (FCSRT-IR).

### Apparatus

For eye-tracking, the EyeLink Desktop 1000 eye-tracker (SR Research) with a sampling rate of 500 Hz was employed. Participants were positioned 55 cm from the monitor (60 Hz), with their dominant eye determined using the Miles test. The EyeLink Desktop 1000 was controlled by the Experiment Builder software (SR Research) to manage stimulus events during eye-tracking tasks.

### Antisaccade Task (AST)

Each AST trial began with a 1-s instruction screen, indicating that the participant should focus on the target. A central fixation target in white on a black background was displayed for one second, followed by a 200-ms blank interval before the appearance of the red saccade distractor. The distractor appeared randomly 4 degrees away from the fixation target, either on the left or right side, for 2 s. Participants were instructed to fixate at the central point and then generate a saccade to the opposite screen position as soon as the distractor appeared. The AST comprised a total of 24 antisaccade trials. There were an additional four practice trials too. The variables obtained from the AST were reaction time (RT) mean and standard deviation (SD). These variables indicate inhibition latency and inhibition latency variability. Antisaccade errors were also computed i.e., the number of incorrect saccades during the AST.

### Prosaccade Task (PST)

The PST followed the same format as the AST, except the participant was told to look toward a target rather than away from the distractor. Each PST trial began with a 1-s instruction screen, indicating that the participant should focus on the target. A central fixation target in white on a black background was displayed for one second, followed by a 200-ms blank interval before the appearance of the green saccade target. The target appeared randomly 4 degrees away from the fixation target, either on the left or right side, for 2 s. Participants were instructed to fixate at the central point and then generate a saccade to the target position as soon as it appeared. The PST comprised a total of 14 antisaccade trials. There were an additional four practice trials too. The variables obtained from the PST were reaction time (RT) mean and standard deviation (SD). These variables indicate prosaccade latency and variability.

### Montreal Cognitive Assessment (MoCA)

The Montreal Cognitive Assessment (Nasreddine et al., 2005) is a brief screening tool for Alzheimer's dementia, assessing attention and concentration, executive functions, memory, language, visuoconstructional skills, conceptual thinking, calculations, and orientation. Scores ranged up to 30, with 26 or more considered normal.

### Free Cued Selective Reminding Task Immediate Recall (FCSRT-IR)

Memory was evaluated with the Free Cued Selective Reminding Task Immediate Recall (FCSRT-IR: Grober & Buschke, 1987). Participants memorised line drawings of recognisable objects (e.g., grapes) with unique category cues (e.g., fruit). The recall process involved presenting cards with 16 items, and participants pointed to and named each item after its cue. A test phase followed, including three recall trials with free and cued recall measures. Free recall scores ranged to 48, with scores below 50% indicating memory impairments. Total scores also ranged to 48, with scores below 90% indicating memory impairments.

### Data Processing

Raw eye-tracking data from EyeLink DataViewer were analysed offline using custom software (Mardanbegi et al., 2019). Noise and spikes were filtered by removing all the frames where the velocity signal was greater than 1,500 degrees/s or the acceleration signal was greater than 100,000 degrees/s^2^. All the fixations and saccadic events were detected by the EyeLink parser, and saccade properties were stored in a table. Microsaccades with an amplitude less than 0.7 degree were filtered out. This filtering step was implemented to minimise the influence of low-amplitude, potentially involuntary eye movements, and to focus on the larger saccadic eye movements associated with voluntary and reflexive attentional shifts. Saccade latency was measured from onset to target onset, including saccades within the 80–700 ms window after target onset to exclude anticipatory saccades (see Boz, 2024; Wilcockson et al., 2019). Participant eye movement metrics over four SD away from the group mean were excluded from analyses. This resulted in one healthy control being excluded from the prosaccade mean RT analyses.

### Statistical Analysis

The overall aim was to compare the performance of individuals with FCD, MCI, and healthy controls on the antisaccade and prosaccade tasks. The primary outcome measures were the reaction time (RT) mean and RT standard deviation (SD) from the trials where participants made correct eye movements, as well as the overall error rate. One-way analyses of variance is performed for each group across the five variables of interest in order to observe broad group differences. To test the hypothesis that individuals with FCD would perform similarly to healthy controls on the antisaccade task, independent *t*-tests were conducted between the FCD and healthy control groups for antisaccade RT mean, RT SD, and error rate. Similarly, to examine whether individuals with FCD would demonstrate prosaccade deficits compared to healthy controls and MCI groups, independent *t*-tests were conducted between the FCD group and both the healthy control and MCI groups for prosaccade RT mean, RT SD, and error rate.

Additionally, a Bayes factor with default prior scales is computed for each analysis, yielding a null result (Love et al., 2019; Morey & Rouder, 2014; Rouder et al., 2012). Bayesian analyses were also conducted to provide additional insights into the strength of evidence for or against the null hypotheses. Computing a Bayes factor provides us with the ability to meaningfully interpret *p*-values > .05. Therefore, a BF10 < 0.33 indicates some evidence for the null hypothesis. BF10 > 3 provides strong evidence for the alternate hypothesis (Rouder et al., 2012).

Prior to conducting the independent *t-*tests, the data for each outcome measure were assessed for normality using Kolmogorov–Smirnov and homogeneity of variance Levene's test. Where the data was found to be non-normally distributed a Mann–Whitney *U* test is reported in place of the independent sample *t-*test and where the data was found to be heteroscedastic and equal variances are not assumed, then a Welch's *t-*test is conducted.

## Results

Firstly, a series of one-way ANOVAs revealed significant group differences for antisaccade RT Mean [*F*(2, 104) = 8.669; *p* < .001], antisaccade RT SD [*F*(2, 104) = 6.838; *p* = .002], antisaccade error rate [%: *F*(2, 106) = 7.882; *p* < .001], prosaccade RT Mean [*F*(2, 96) = 3.772; *p* = .027], and prosaccade RT SD [*F*(2, 97) = 3.290; *p* = .042]. It was then confirmed that the MCI group differed significantly from healthy controls in terms of antisaccade RT mean [*t*(81) = 3.866; *p* < .001], antisaccade RT SD [*t*(81) = 3.508; *p* < .001] and antisaccade error rate (*U* = 1092; *p* = .020), in all cases indicating the MCI group were impaired compared to the controls. Interestingly, the FCD group did not differ significantly from healthy controls for either antisaccade RT mean [*t*(74) = .688; *p* = .494; BF10 = .315], antisaccade RT SD [*t*(74) = .928; *p* = .356; BF10 = .371], nor antisaccade error rate (*U* = 556; *p* = .666; BF10 = .276) but FCD did differ significantly from MCI antisaccade RT mean [*t*(49) = 3.033; *p* = .004], antisaccade RT SD [*t*(49) = 2.234; *p* = .030], and antisaccade error rate (*U* = 468; *p* = .022), indicating that the MCI group were significantly more impaired than the FCD group. These results may indicate that FCD performs more similarly to controls than they do to MCI patients on the antisaccade task (see Table 2).

**Figure 1.**
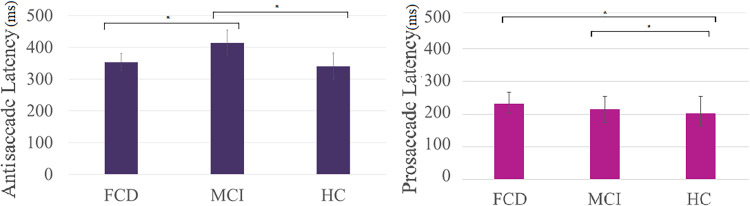
Graphs demonstrating how the different groups are differentiated by the different saccade tasks. MCI differs from both FCD and HC in terms of antisaccade latency (ms) whereas HCs and FCDs differ in terms of prosaccade latency (ms) and MCI and HCs differ in terms of prosaccade latencies (ms) too.

Analyses were then conducted on the prosaccade task. It was found that the MCI group did not differ significantly from healthy controls in terms of prosaccade RT mean [*t*(80) = 1.060; *p* = .293; BF10 = .500] nor prosaccade RT SD [*t*(81) = 1.484; *p* = .142; BF10 = .759]. This provides evidence that MCI patients may have intact prosaccade eye movements. Interestingly however, the FCD group were significantly more impaired that the healthy controls for prosaccade RT mean [*t*(85) = 2.578; *p* = .012] and prosaccade RT SD [*t*(86) = 2.329; *p* = .022], but despite the lack of significant difference between MCI and healthy controls and the difference between FCD and healthy controls, FCD surprisingly did not differ significantly from MCI prosaccade RT mean [*t*(20.87) = −1.166; *p* = .257; BF10 = .542] and prosaccade RT SD [*t*(23) = −.308; *p* = .761; BF10 = .386]. These results may indicate that FCD may differ from healthy controls in terms of prosaccade performance, but their prosaccade performance may not be too distinct from MCI (see Figure 1).

Finally, correlations between the saccadic parameters and cognitive performance variables for the patients with FCD and MCI, as well as HCs are shown in Table 3. It was revealed that the MoCA was associated with performance on antisaccade RT SD for FCD and MCI, antisaccade error rate for MCI, and antisaccade RT mean for HCs. The FCSRT free recall was associated with antisaccade RT mean in HCs, and the FCSRT total score was associated with antisaccade RT mean and antisaccade error rate in the HCs. Neither the prosaccade measure was associated with any of the cognition scores. Overall, these results indicate that within-groups associations between eye movement measures and cognitive scores were sparse and inconsistent. There was a trend for antisaccade performance to be associated with MoCA score, but this was not consistent across all groups.

**Table 3.** Correlations between saccadic parameters and cognitive performance variables.

		Antisaccade RT mean	Antisaccade RT SD	Antisaccade error rate (%)	Prosaccade RT mean	Prosaccade RT SD
MoCA total score	FCD	.054	−.517*	−.300	−.114	−.210
	MCI	−.085	−.503*	−.545*	−.245	−.269
	HC	−.344*	−.089	−.190	.182	.196
FCSRT – free recall	FCD	.211	−.281	−.253	−.017	.028
	MCI	−.044	−.245	−.433	.118	−.059
	HC	−.368*	−.169	−.279	.392	.038
FCSRT − total	FCD	−.113	−.242	−.033	−.462	.030
	MCI	.131	.065	.037	−.092	−.596
	HC	−.532*	−.255	−.545*	−.134	.271

*Note*. Significant differences between groups are denoted by asterisk “*”.

## Discussion

The present study aimed to investigate the utility of antisaccade and prosaccade eye movement tasks in differentiating individuals with FCD from those with MCI and healthy controls. The findings provide intriguing insights into the potential role of eye movements as objective markers in the assessment of functional cognitive complaints. The first hypothesis, suggesting that individuals with FCD would exhibit performance on antisaccade tasks comparable to healthy controls, was supported. FCD participants did not significantly differ from healthy controls in antisaccade reaction time mean or standard deviation. This finding implies that the inhibitory control of saccadic eye movements, a process primarily associated with intact executive functions, appears to be preserved in individuals with FCD, aligning with the functional nature of their cognitive complaints. Interestingly, FCD participants exhibited antisaccade performance more similar to healthy controls than to MCI patients, suggesting a potential distinct profile in eye movement performance for FCD. Contrastingly, the second hypothesis proposes that individuals with FCD would demonstrate prosaccade deficits not present in either the MCI or healthy control groups was partially supported. FCD participants differed significantly from healthy controls in prosaccade reaction time mean and standard deviation, indicating altered performance in reflexive bottom-up eye movements. However, the lack of significant differences between FCD and MCI in prosaccade performance suggests a more nuanced relationship. The discrepancy between FCD and MCI in prosaccade tasks may reflect the complex interplay between conscious compensatory mechanisms and cognitive processes implicated in bottom-up attentional control.

The findings demonstrate preserved antisaccade performance observed in individuals with FCD aligns with the notion that these individuals might employ compensatory strategies to consciously monitor and adjust for perceived lapses in attention (Pennington et al., 2015; Stone et al., 2015). While this compensatory effort maintains inhibitory control in antisaccade tasks, it may result in altered performance in prosaccade tasks, reflecting a potential impact on reflexive bottom-up attentional processes. The analyses provide evidence suggesting that individuals with suspected FCD may exhibit delayed prosaccade performance while maintaining intact antisaccade performance. This pattern differs from that observed in MCI patients, who exhibit intact prosaccade but impaired antisaccade (Wilcockson et al., 2019). This contrast suggests that MCI patients may face challenges specifically related to inhibitory control in eye movement saccades, a difficulty not shared by FCD patients. Additionally, MCI patients are not impaired in prosaccade latency (RT and SD), unlike FCD patients, indicating a distinction in their abilities. This suggests that FCD patients may experience delayed prosaccades, aligning with theories associating FCD with more deliberate and controlled attention. The results imply that patients with FCD maintain intact top-down processing of visual stimuli, whereas their bottom-up processing of stimuli is impaired, resulting in delayed voluntary eye movements. Consequently, these findings suggest that rumination about cognitive decline may impact bottom-up processing due to the conscious monitoring of attentional lapses. While the cause of increased rumination about cognitive decline is beyond the scope of this paper, given the absence of issues attributable to brain injury or disease, the observed delay in attentional processes is likely functional and, therefore, may be addressed with appropriate intervention.

The study may ultimately have implications for clinical practice. Although further research is still needed, eye movement tasks like the prosaccade and antisaccade may have potential to one day serve as a valuable addition to traditional diagnostic methods in Memory Clinics, with the antisaccade showing potential to measure cognitive decline whilst the prosaccade potentially measuring functional issues. The observed dissociation in eye movement profiles between FCD, MCI, and healthy controls suggests that eye tracking may contribute to the differentiation of these conditions, aiding clinicians in more accurate and efficient diagnostic processes. It was observed that patients with FCD have a specific pattern of eye movements distinct from patients with more objective cognitive impairment. Previously FCD may have been diagnosed through a process of elimination of other conditions; however, with further research into specificity and sensitivity, the distinct pattern of eye movements observed in this paper could eventually be utilised as a “positive sign” in clinical settings to make a “probable diagnosis” of FCD. Further, the inconsistency within-group correlations between eye movement parameters and cognitive scores suggests that global cognitive measures may not fully capture the specific cognitive processes measured by these oculomotor tasks. This discrepancy highlights the potential for eye-tracking to provide unique information about cognitive function, distinct from that obtained through traditional neuropsychological testing. However, an important next step would be a large multi-centre trial to confirm that the antisaccade test is able to reliably diagnose early AD. This would then facilitate endorsement by professional bodies and clinical evaluation.

Despite the promising results, several limitations should be acknowledged. The relatively small sample size in the FCD group may limit the generalisability of the findings. Additionally, the ethnic homogeneity of the sample lowers generalisability. Future research with larger and more diverse samples is warranted to further validate the observed patterns. Additionally, a weakness of the study is how the groups were defined. The study relied upon the assessment of elderly people in memory clinics. Although no impairments in activities of daily living were reported, as this is an elderly sample, it may be that the FCD group is an earlier stage of MCI. However, it should be noted that the FCD sample had no objective memory impairment, only subjective, and previous research indicates that subjective memory impairment is not always a precursor to objective cognitive decline. However, the cross-sectional nature of the study prevents the examination of longitudinal changes in eye movement patterns in FCD.

In conclusion, this study provides preliminary evidence for the utility of eye movements as potential objective markers to distinguish individuals with FCD from those with MCI and healthy controls. This promising insight may pave the way for the development of more effective diagnostic tools and a deeper understanding of the potentially functional nature of cognitive complaints in FCD.

## References

[bibr1-03010066251359215] Ball H. A. McWhirter L. Ballard C. Bhome R. Blackburn D. J. Edwards M. J. Fleming S. M. Fox N. C. Howard R. Huntley J. Isaacs J. D. Larner A. J. Nicholson T. R. Pennington C. M. Poole N. Price G. Price J. P. Reuber M. Ritchie C. , …, Carson A. J. (2020). Functional cognitive disorder: Dementia’s blind spot. Brain: A Journal of Neurology, 143, 2895–2903. 10.1093/brain/awaa224 32791521 PMC7586080

[bibr2-03010066251359215] Borelli W. V. de Senna P. N. Brum W. S. Schumacher-Schuh A. F. Zimmer E. R. Fagundes Chaves M. L. Castilhos R. M. (2022). Functional cognitive disorder presents high frequency and distinct clinical profile in patients with low education. Frontiers in Aging Neuroscience, 14, 789190. 10.3389/fnagi.2022.789190 35431909 PMC9011344

[bibr3-03010066251359215] Boz E. (2024). Uncorrected errors and correct saccades in the antisaccade task distinguish between early-stage Alzheimer’s disease dementia, amnestic mild cognitive impairment, and normal aging. Aging, Neuropsychology, and Cognition (Neuropsychology, Development and Cogniti, 31, 457–478. 10.1080/13825585.2023.2198191 37004192

[bibr4-03010066251359215] Cabreira V. Frostholm L. McWhirter L. Stone J. Carson A. (2023). Clinical signs in functional cognitive disorders: A systematic review and diagnostic meta-analysis. Journal of Psychosomatic Research, 173, 111447. 10.1016/j.jpsychores.2023.111447 37567095

[bibr5-03010066251359215] Grober E. Buschke H. (1987). Genuine memory deficits in dementia. Developmental Neuropsychology, 3, 13–36. 10.1080/87565648709540361

[bibr6-03010066251359215] Hallett M. Aybek S. Dworetzky B. A. McWhirter L. Staab J. P. Stone J. (2022). Functional neurological disorder: New subtypes and shared mechanisms. The Lancet Neurology, 21, 537–550. 10.1016/S1474-4422(21)00422-1 35430029 PMC9107510

[bibr7-03010066251359215] Ishihara S. M. (1983). Ishihara's tests for colour-blindness. Kanehara and Co.

[bibr8-03010066251359215] Jessen F. Amariglio R. E. Buckley R. F. van der Flier W. M. Han Y. Molinuevo J. L. Rabin L. Rentz D. M. Rodriguez-Gomez O. Saykin A. J. Sikkes S. A. M. Smart C. M. Wolfsgruber S. Wagner M. (2020). The characterisation of subjective cognitive decline. The Lancet Neurology, 19, 271–278. 10.1016/S1474-4422(19)30368-0 31958406 PMC7062546

[bibr9-03010066251359215] Kirova A.-M. Bays R. B. Lagalwar S. (2015). Working memory and executive function decline across normal aging, mild cognitive impairment, and Alzheimer’s disease. BioMed Research International, 2015, 748212. 10.1155/2015/748212 26550575 PMC4624908

[bibr10-03010066251359215] Love J. Selker R. Marsman M. Jamil T. Dropmann D. Verhagen J. Ly A. Gronau Q. F. Smíra M. Epskamp S. Matzke D. Wild A. Knight P. Rouder J. N. Morey R. D. Wagenmakers E.-J. (2019). JASP: Graphical Statistical Software for Common Statistical Designs. Journal of Statistical Software, 88, 1–17. 10.18637/jss.v088.i02

[bibr11-03010066251359215] Mardanbegi D. Wilcockson T. Sawyer P. Gellersen H. Crawford T. (2019). SaccadeMachine: Software for analyzing saccade tests (anti-saccade and pro-saccade). Proceedings of the 11th ACM Symposium on Eye Tracking Research & Applications (pp. 1–8). 10.1145/3317956.3318148

[bibr12-03010066251359215] McWhirter L. Ritchie C. Stone J. Carson A. (2020). Functional cognitive disorders: A systematic review. The Lancet. Psychiatry, 7, 191–207. 10.1016/S2215-0366(19)30405-5 31732482

[bibr13-03010066251359215] Morey R. D. Rouder J. N. (2014). *BayesFactor (Version 0.9.10-2). Computer Software. Retrieved from* http://bayesfactorpcl.r-forge.r-project.org/.

[bibr14-03010066251359215] Munoz D. P. Everling S. (2004). Look away: The anti-saccade task and the voluntary control of eye movement. Nature Reviews. Neuroscience, 5, 218–228. 10.1038/nrn1345 14976521

[bibr15-03010066251359215] Nasreddine Z. S. Phillips N. A. Bédirian V. Charbonneau S. Whitehead V. Collin I. Cummings J. L. Chertkow H. (2005). The Montreal cognitive assessment, MoCA: A brief screening tool for mild cognitive impairment. Journal of the American Geriatrics Society, 53, 695–699. 10.1111/j.1532-5415.2005.53221.x 15817019

[bibr16-03010066251359215] Opwonya J. Doan D. N. T. Kim S. G. Kim J. I. Ku B. Kim S. Park S. Kim J. U. (2022). Saccadic eye movement in mild cognitive impairment and Alzheimer’s disease: A systematic review and meta-analysis. Neuropsychology Review, 32, 193–227. 10.1007/s11065-021-09495-3 33959887 PMC9090874

[bibr17-03010066251359215] Pennington C. Hayre A. Newson M. Coulthard E. (2015). Functional cognitive disorder: A common cause of subjective cognitive symptoms. Journal of Alzheimer’s Disease: JAD, 48, S19–S24. 10.3233/JAD-150182 26402086

[bibr18-03010066251359215] Perry R. J. Hodges J. R. (1999). Attention and executive deficits in Alzheimer’s disease. A critical review. Brain: A Journal of Neurology, 122, 383–404. 10.1093/brain/122.3.383 10094249

[bibr19-03010066251359215] Petersen R. C. (2004). Mild cognitive impairment as a diagnostic entity. Journal of Internal Medicine, 256, 183–194. 10.1111/j.1365-2796.2004.01388.x 15324362

[bibr20-03010066251359215] Rouder J. N. Morey R. D. Speckman P. L. Province J. M. (2012). Default Bayes factors for ANOVA designs. Journal of Mathematical Psychology, 56, 356–374. 10.1016/j.jmp.2012.08.001

[bibr21-03010066251359215] Silverberg N. D. Rush B. K. (2024). Neuropsychological evaluation of functional cognitive disorder: A narrative review. The Clinical Neuropsychologist, 38, 302–325. 10.1080/13854046.2023.2228527 37369579

[bibr22-03010066251359215] Stone J. Pal S. Blackburn D. Reuber M. Thekkumpurath P. Carson A. (2015). Functional (psychogenic) cognitive disorders: A perspective from the neurology clinic. Journal of Alzheimer’s Disease: JAD, 48, S5–S17. 10.3233/JAD-150430 26445274

[bibr23-03010066251359215] Teodoro T. Edwards M. J. Isaacs J. D. (2018). A unifying theory for cognitive abnormalities in functional neurological disorders, fibromyalgia and chronic fatigue syndrome: Systematic review. Journal of Neurology, Neurosurgery, and Psychiatry, 89, 1308–1319. 10.1136/jnnp-2017-317823 29735513 PMC6288708

[bibr24-03010066251359215] Van Patten R. Bellone J. A. (2023). The neuropsychology of functional neurological disorders. Journal of Clinical and Experimental Neuropsychology, 45(10), 957–969. 38441076 10.1080/13803395.2024.2322798

[bibr25-03010066251359215] Wakefield S. J. Blackburn D. J. Harkness K. Khan A. Reuber M. Venneri A. (2018). Distinctive neuropsychological profiles differentiate patients with functional memory disorder from patients with amnestic-mild cognitive impairment. Acta Neuropsychiatrica, 30, 90–96. 10.1017/neu.2017.21 28714423

[bibr26-03010066251359215] Wilcockson T. D. W. Mardanbegi D. Xia B. Taylor S. Sawyer P. Gellersen H. W. Leroi I. Killick R. Crawford T. J. (2019). Abnormalities of saccadic eye movements in dementia due to Alzheimer’s disease and mild cognitive impairment. Aging, 11, 5389–5398. 10.18632/aging.102118 31375642 PMC6710064

